# *In Silico* Screening, Alanine Mutation, and DFT Approaches for Identification of NS2B/NS3 Protease Inhibitors

**DOI:** 10.1155/2016/7264080

**Published:** 2016-02-25

**Authors:** R. Balajee, V. Srinivasadesikan, M. Sakthivadivel, P. Gunasekaran

**Affiliations:** ^1^Medicinal Chemistry Group, Institute of Chemistry of Sao Carlos, University of Sao Paulo, Sao Carlos, Brazil; ^2^Department of Applied Chemistry, National Chiao Tung University, Hsinchu, Taiwan; ^3^King Institute of Preventive Medicine, Guindy, Chennai, India

## Abstract

To identify the ligand that binds to a target protein with high affinity is a nontrivial task in computer-assisted approaches. Antiviral drugs have been identified for NS2B/NS3 protease enzyme on the mechanism to cleave the viral protein using the computational tools. The consequence of the molecular docking, free energy calculations, and simulation protocols explores the better ligand. It provides in-depth structural insights with the catalytic triad of His51, Asp75, Ser135, and Gly133. The MD simulation was employed here to predict the stability of the complex. The alanine mutation has been performed and its stability was monitored by using the molecular dynamics simulation. The minimal RMSD value suggests that the derived complexes are close to equilibrium. The DFT outcome reveals that the HOMO-LUMO gap of Ligand**19** is 2.86 kcal/mol. Among the considered ligands, Ligand**19** shows the lowest gap and it is suggested that the HOMO of Ligand**19** may transfer the electrons to the LUMO in the active regions. The calculated binding energy of Ligand**19** using the DFT method is in good agreement with the docking studies. The pharmacological activity of ligand was performed and satisfies Lipinski rule of 5. Moreover, the computational results are compared with the available IC_50_ values of experimental results.

## 1. Introduction

Dengue virus (DV, DENV 1–4) belongs to Flaviviridae family; infection regardless of its serotypes is transmitted from person to person by the female leads of* Aedes aegypti* or* Aedes albopictus* mosquitoes in the domestic environment and causes a serious public health issues across the globe [1, 2]. Recently, two thousand cases were reported in Portugal and many cases were detected in European part and many cases in Asia [3]. It is one of the most prevalent arthropod-borne viral diseases in terms of morbidity and mortality [4].

An integral membrane protein of NS2B and NS3 (NS2B/NS3) in the serine protease enzyme is essential for viral replication [5]. The structural insight shows that NS3 belongs to a second larger protein and contains the catalytic triad and is colocalized within distinct paracrystalline of convoluted membranes [6]. It is termed as crucial because it is being matured with the presence of cofactor NS2B and enables the polyprotein posttranslationally in endoplasmic reticulum [7, 8]. The processing occurs in P1 and P2 positions, where P2 position carries Lys-Arg, Arg-Arg, and Arg-Lys and oftenly P1 position carries Gln-Arg followed by Gly, Ala, or Ser. Therefore, the biologically active viral protease of NS2B/NS3 complex is termed as heterodimeric [9]. The hydrophobic regions are associated with the polyprotein precursor, which regulates the protease sensitive sites in the optimal context of* cis*- and* trans*-cleavages. Therefore, the biologically active viral protease of NS2B/NS3 complex is termed as heterodimeric [9].

Earlier reports states that the various inhibitors from the different sources are analyzed using* in vitro* and* in silico* approaches to determine their biological activity and binding modes with close proximity residues. The active site was detected in the major surface of the C-terminal domain region and substrate binds to the polyprotein sequences to carry out the cleavage process [10]. The series of inhibitors like panduratin, hydroxypanduratin, and so forth have been explored from the molecular docking analysis. The key residue responsible for interaction between the protein and inhibitors was established [11]. The alanine substitution detects the precleavage at NS2B/NS3 complex and may cause the proteolytic activity to decline [12]. The mutation in complex causes the proteolytic activity to decline [13]. Hence, the proposed complex is analyzed to be a promising target in the search for drugs against DV.

**Figure 1:**
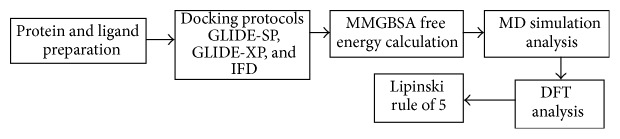
Schematic representation of proposed research.

Frontiers in the drug discovery research challenges the pharmaceutical industries [14]. So, the scientific community is taking more efforts to discover the therapeutic target towards the NS2B/NS3 proteins. Currently, the work is designed to identify the novel potential inhibitor for the NS2/NS3 protease enzyme for dengue virus. The study was observed from various computational protocols like molecular docking, molecular dynamics simulation, free energy calculations, and DFT approaches and the flow chart is shown in Figure 1. MD simulations were performed to determine the stability and dynamical changes of predicted binding conformation and analysis extended between Wild Type (WT) and mutated consequences. A MM-GB/SA analysis was carried out to calculate the binding free energies of the complex and to determine the characteristics of drug-like molecule towards the NS2/NS3 protease enzyme. Moreover, the* state-of-the-art* density functional theory (DFT) investigations have also been carried out at M06 [15] level with the 6-31g(d) basis set to better understand the hydrogen bonding strengths, binding energy, and the interaction sites of amino acids with the ligands at molecular level. The properties of drug-like molecule like hydrogen bond, physicochemical parameters of Lipinski rule of 5 (RO5) [16], and the results from this study will be highly useful for further investigations.

## 2. Materials and Methods

The Schrodinger molecular modelling suite was employed to carry out the molecular docking, dynamics simulation analysis, and free energy estimations. To determine the hydrogen bonding strengths, molecular orbitals (HOMO-LUMO), electrostatic potential (ESP), and binding energy between ligand selected amino acids the density functional theory calculations have been carried out by using Gaussian 09 program [17]. Due to the computational limitation we have considered only two to three key amino acids which are interacted with the ligands for the DFT study. And we have taken four ligands for the DFT investigation which have shown the higher binding energy and scoring function in the docking studies. The further DFT study has been carried out to understand the key amino acids in the binding pocket interacting with the ligands at molecular level. The geometries are optimized at M06/6-31g(d) level of theory in gas phase. Molecular orbitals are visualized using Gauss view 5.0.8. The binding energies are calculated using the following formula:

(1) Binding  EnergyBE=ELigand-Amino  acids−ELigand+EAmino  acids.

And the electrostatic potential (ESP) calculations have been carried out using the fchk and pop, regular key words in Gaussian 09.

### 2.1. Ligand Background

The binding affinity between the receptor and inhibitor determines the biological activity; it was influenced by geometrical positions and steric and physical properties. The distinct of structures was explored and leads to the large set of biological diversities. For the investigation, the ligands were obtained from Pubchem/Drugbank and Chemdiv as shown in Table 1 [10]. The structure of these inhibitors along with binding energies and IC_50_ values was given in Table 1.

### 2.2. Preparation Methodologies

A diverse set of ligands were obtained from PubChem [18], DrugBank [19], and Chemical Diversity, USA [20, 21]. Each ligand was subjected to a minimization with the OPLS2005 force field to eliminate the steric clashes of bond length and angles in the crystal structure and prepared with pH 7.0 using LigPrep module [22, 23]. The structural coordinates of dengue virus NS2B/NS3 protease were extracted from the Protein Data Bank (PDB ID: 2fom) [24] and structure was minimized using IMPACT module with steepest descent gradient algorithm for about 100 cycles [25]. The structure was optimized using protein preparation wizard in a similar fashion [26].

### 2.3. Docking Protocol

GLIDE parameters were applied with VdW scaling of 0.8 and partial cut-off of 0.15 was implemented to soften the potential for nonpolar sites and no constraints were specified. The grid box was generated according to the residues suggested with the VdW scaling factor of 0.8 and partial cut-off of 0.15 [27]. The ligands were docked using the “standard precision” (GLIDE-SP) and “extra precision” (GLIDE-XP) [28]. Firstly, the ligands were screened using GLIDE-SP and the GLIDE-XP approach was executed to ensure the good score and poses. The outcome was generated according to the GLIDE Energy and interactions [29]. The least energy values were selected for further analysis.

## 3. Induced Fit Docking (IFD) Protocol

The outcome of GLIDE-XP was considered to perform the IFD protocol and this is termed as second phase. This is considered as a flexible protein with flexible ligand. The resulting ligands were docked individually using the following steps: (a) the receptor was defined with the aforementioned active site residues and (b) in the initial GLIDE docking stage soften potential docking with the van der Waals radii scaling of 0.50 [30] for the protein was performed to retain the maximum number of 5 poses per ligand.

## 4. MMGBSA Protocol

The Prime-MM/GBSA protocol was employed to predict the free energy of binding complexes. The complex was minimized with the OPLS-2005 Force Field [31] Prime which uses the VSGB model employing a surface representation of the Solvent Accessible Surface Area [32].

## 5. Dynamics Calculations

MD calculations were performed using Desmond version 2.0, Schrodinger LLC. The best outcome of IFD was initiated with TIP3P water model and orthorhombic box buffer size of 10 Å and the system was neutralized with 0.15 M using Na^+^ ions. Force field parameters of the protein-ligand systems are assigned with OPLS-2005, constrained using the SHAKE algorithm with the period boundary conditions (PBC), and electrostatic interactions were applied using the Particle Mesh Ewald (PME) method [33]. For the dynamics, a multistep RESPA integration algorithm was used throughout 1.2 fs. The relaxation was followed by 10 ns production run in the NPT ensemble with temperature 300 K, thermostat relaxation time 1.0 ps, and barostat relaxation time 2.0 ps which was performed for each system using a Nose-Hoover thermostat and Martyna-Tobias-Klein barostat and prepared with similar fashion for the period of 10 ns [34].

### 5.1. Mutational Analysis

The alanine was substituted with the following residues Leu115, Asp129, Gly133, Thr134, Ser163, and Ile165 which are suggested in order to determine the protein stability [35]. The docking and simulation interpretation was performed with the aforementioned methodologies.

## 6. Results and Discussions

### 6.1. Docking Analysis

**Figure 2:**
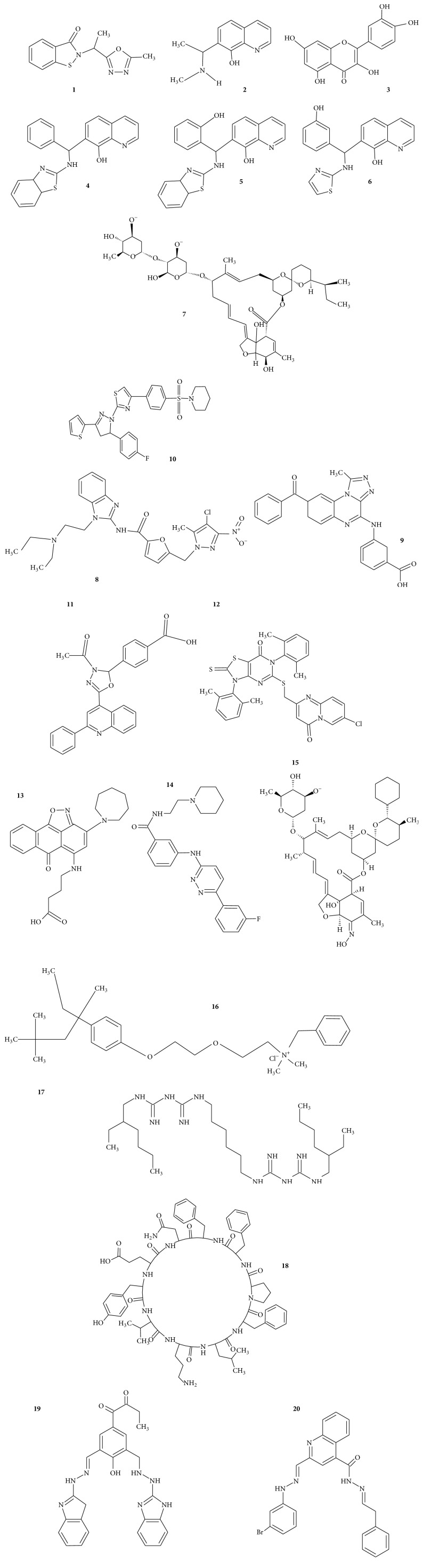
Top ranked 20 ligand structures. Ligand numbers only provided near to each ligand structure.

**Figure 3:**
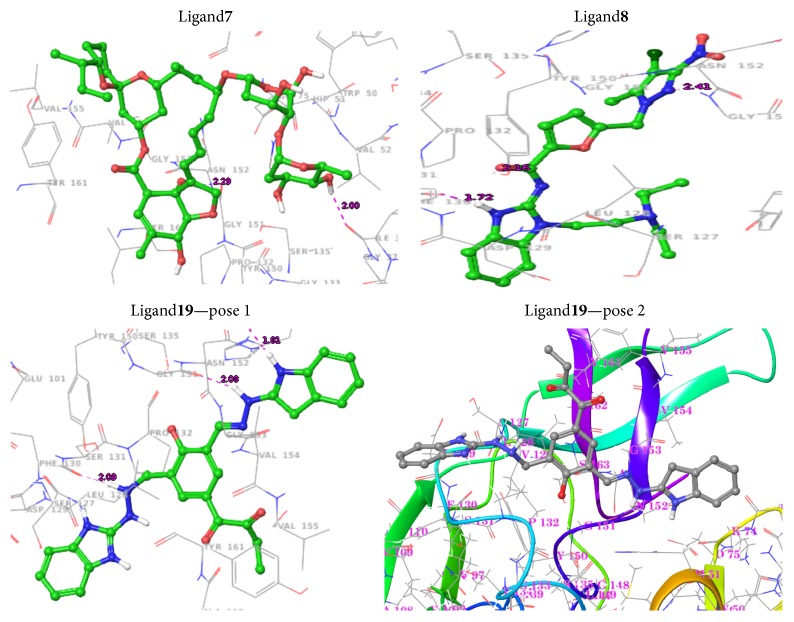
Interactions of ligand-protein complexes.

The region of NS2B protein associated with NS3 protein has greater impact to enhance their activity [36]. The screened ligands (Figure 2) categorized according to the energy values reported in Table 1 and the respective hydrogen bond interactions are shown in Figure 3. The result reveals that Ligand**7**, Ligand**8**, Ligand**14**, and Ligand**19** are much closer towards active site. These ligands are further analyzed with Induced Fit Docking approach.

### 6.2. Induced Fit Docking

Induced Fit Docking (IFD) approach was aimed to detect the conformational changes of the protein and to study the characteristics of the ligands. The analysis was performed from the outcome of the GLIDE-XP. The IFD result reveals the better poses in contrast to the more poses generated for the following ligands: Ligand**7**, Ligand**8**, and Ligand**19**, and the least energy values −49.17, −62.71, and  −64.16, respectively, reported in Table 2. Ligand**19** of pose 2 has a better interaction with the catalytic site given in Table 3 and exhibits realistic energy value to have a property of drug-like molecule. The outcome of IFD was advanced to estimate the binding affinity in the free energy state.

## 7. MM-GBSA Docking

**Figure 4:**
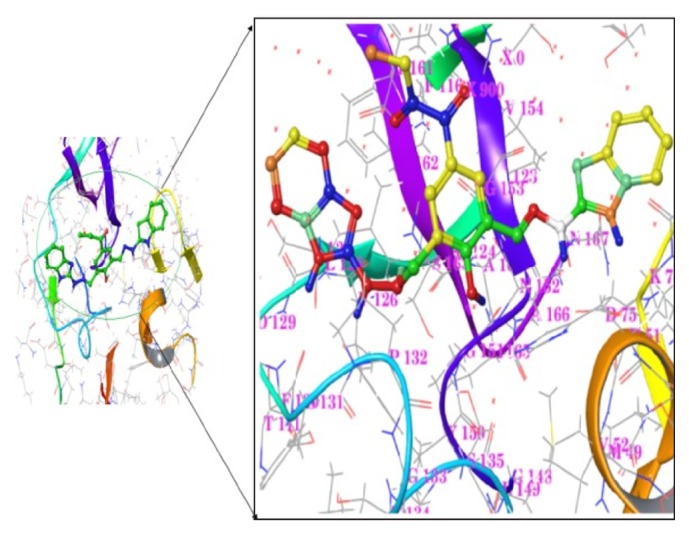
A close-up view representation of docked complexes from MM-GBSA analysis. The ligand maintains the direct contact with Phe130, Gly153, Gly151, and Asp75 indicated in the circled regions and zoomed on with their nearest residues indicated.

The calculated free energy complex ranges from −53 to  −74 Kcal/mol shown in Table 4. According to the MMGBSA results, the major favourable contributions to the ligand binding are VdW (Δ*G*_vdw_) and solvation terms (Δ*G*_sol_). These results provide more significance towards the binding affinity depicted in Figure 4. Therefore, this study describes that *G*_solv_ is an important factor to steer the force for ligand binding.

The docking protocol was evaluated in order to identify the distance between the active site residues and ligands targeted between 1.7 Å and 2.5 Å. The following insights were observed from the earlier report of Murthy et al. and Kee et al. [37, 38]; the Gly133 and Ser135 were actively contributed in oxyanion hole and the catalytic triad was generated with the residues of His51, Asp75, and Ser135 which incorporated in the active site regions. In this context, the interactions are being considered as main criteria; Ligand**19** seems to be a potential and interacted with the least distance to the following residues: Asp75, Gly151, and Gly153, when compared with existing ligands like panduratin and hydroxypanduratin [11] shown in Table 5. It shows a better interaction with respect to the distances with existing residues as well as established interaction with Phe130 provided as the stable contact shown in Figure 4.

The strong interactions N-H⋯O and O-H⋯O are more crucial towards the protein-ligand binding [39]. At this point, the reported ligand having the direct contact about the distance of ~2.4 Å with the active regions, especially carboxyl oxygen group of Phe130, is targeted towards amine group of the ligand as it may be involved in electrostatic forces. Gly151 and Asp75 residue of carboxylic oxygen group have a reasonable contact with amine group of the ligand. In another site, Gly153 of carboxyl group has an interaction with the amine group of the ligand. The results reveal the stability by displaying more contacts in the form of N-H⋯O. Pertaining to this investigation, the ligand resided in the binding pocket with the greater impact on interactions to play the role of a drug-like character and makes the oxyanion hole and the catalytic triad more active. The identified ligand molecule has a good agreement with the earlier reports [11, 39] and can be considered as an inhibitor against the dengue virus NS3 protease.

Our results are in good agreement with the previous report which shows that the aforementioned residues His51, Asp75, Gly133, Ser135, Gly151, Asn152, and Gly153 are located in the active site regions and are crucial to the ligand interactions [21, 40]. Moreover, Ser131 has been contributed in VdW interactions revealed from the docking analysis.

## 8. Simulation Analysis

**Figure 5:**
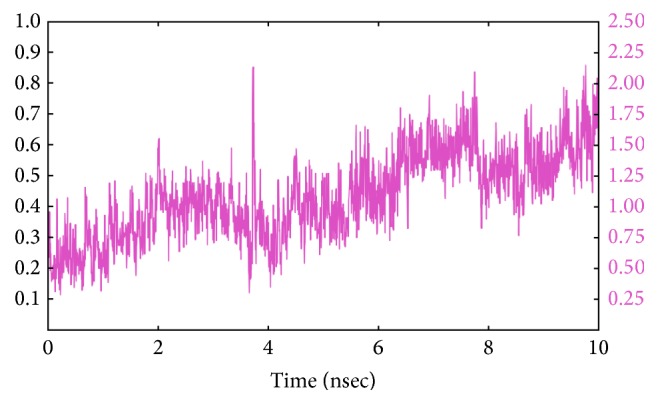
Simulation analysis of NS2/NS3B protease with Ligand**19**.

Figure 5 represents the simulation analysis of the docked complex which is relatively consistent during 10 ns simulation run observed from the RMSD values. The hydrogen bond interactions and close proximity residues stabilized the complex, which results in the minimal RMSDs. The stretch of the RMSD peak fluctuates about 0.5 Å and is boosted immediately to find its convergence after the slight fluctuations. The period between 7 and 10 ns seems to be crucial over stability and the fluctuation occurring at 8.5A does not have a remarkable impact over the stability. Hence, this molecular dynamics simulation shows the significance towards the protein-ligand complex.

## 9. Alanine Screening

**Figure 6:**
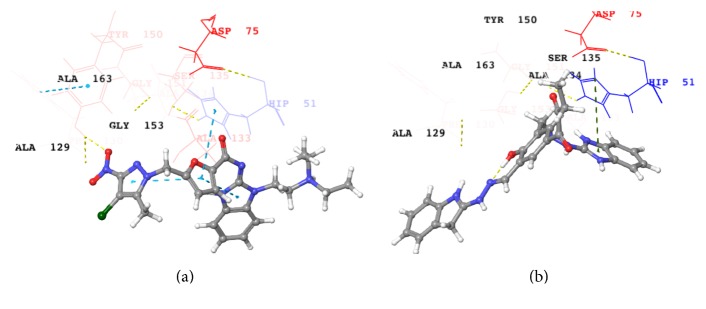
Interaction of ligands with mutated proteins: (a) Ligand**08** and (b) Ligand**19**.

*Alanine Screening*—*Binding Analysis*. This hypothesis explored the mutational action of alanine in the Wild Type (WT) protein with the following residues: Leu115, Asp129, Gly133, Thr134, Ser163, and Ile165. The best docked ligands, Ligand**08** and Ligand**19**, were screened with the mutated protein. Their interactions with the residues remain the same as represented in Figures 6(a) and 6(b).

Salaemae et al. [41] and Robin et al. [42] suggested that the residue Gly133 is a part of conserved motif in NS3 sequences and plays a critical role in oxyanion hole in order to ligand bind. The docking analysis reveals that alanine substitution of Gly133 residue disappears from the closer contact and the rate of the chemical reaction may be degraded [41]. The analysis of residues S163A and I165A has shown their distinguishable significance by changing their conformations and it may also affect the enzymatic activity [43]. The side chain of His51, chain of His51, a positive charge stabilized by the cyclopentyl ring of Ligand**08** and Ligand**19**, was strongly supported by the carboxyl group Asp75 by obtaining the *π*-*π* interactions. Further, Gly151 has a crucial role in form of stability from two aspects: (a) strengthening the tetrahedral transition state towards Ser135 during the substrate cleavage and (b) sustaining the stability of the protease fold [35]. Apart from the aforementioned residues the Leu115, Asp129, and Thr134 have not been involved in the drastic conformational change [41].

**Figure 7:**
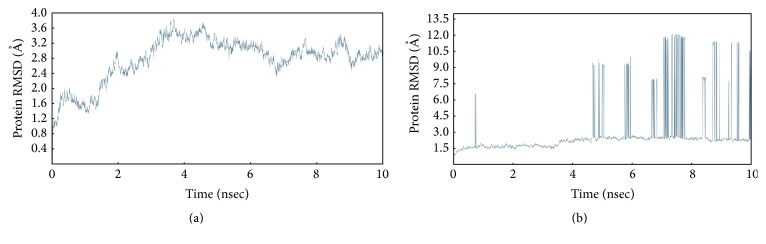
MD simulation of (a) WT protein and (b) alanine mutated protein.

MD simulation analysis which provides an evidence of having a wide change between the WT and mutated proteins is represented in Figure 7(a). The WT protein has initiated their activity within a short time and shown the constant stability of RMSD values between 2.8 and 3.2 Å. The plateau has shown sharp fluctuation at 7.5 ns and it was converged at the end, whereas in mutated protein most of the residues settled between 1.5 and 3.0 Å and some of the residues have shown a drastic change by specifying the plateau peaked up to 12 Å which is shown in Figure 7(b). This indicates that alanine has changed the protein conformation and may disrupt the stability [44].

Our finding shows that dengue virus NS2B/NS3 protease is essentially in agreement with previous data; the molecular simulation studies confirmed that WT protein seems to be stable and mutated protein leads to a change in conformations due to alanine substitution [43, 44]. It demonstrates that alanine substitution did not produce any remarkable effect on proteolytic cleavage [45]. The study clearly indicates the outcome of binding affinity and the simulation analysis of the mutated protein could not be able to carry out further investigations. We believe that the details provided about the designed molecule with Wild Type protein may be sufficient to execute further experimental investigations.

## 10. DFT Analysis

**Figure 8:**
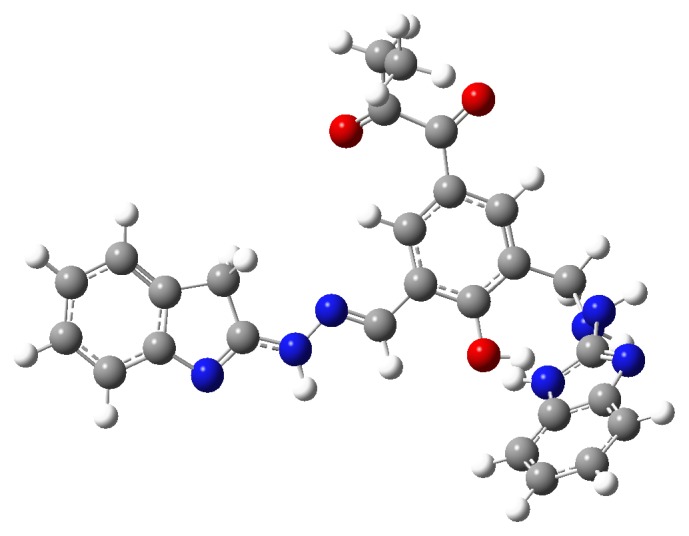
Most stable structure of Ligand**19** in the gas phase optimized at M06/6-31g(d) level in Gaussian 09.

**Figure 9:**
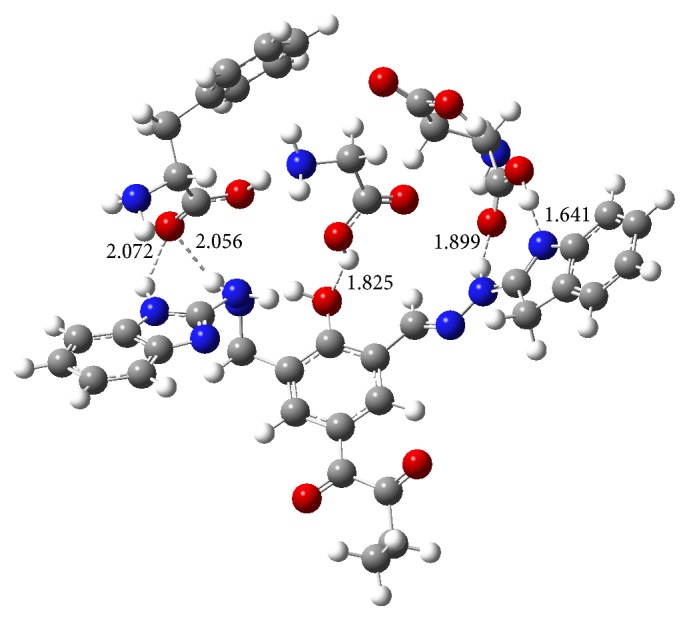
Optimized structure of Ligand**19-**amino acids at M06/6-31g(d) level in gas phase. The hydrogen bond lengths (Å) are noted.

**Figure 10:**
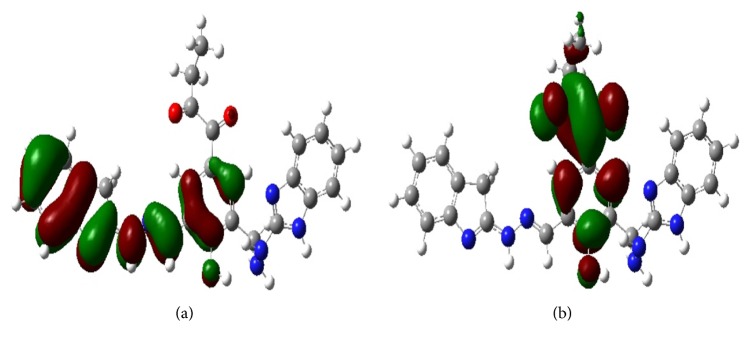
Molecular orbital distribution plots of (a) HOMO and (b) LUMO states in the ground state of Ligand**19** at M06/6-31g(d) level in gas phase.

The DFT calculations have been carried out for the ligands which have obtained the large scoring in the docking studies. For the DFT studies maximum one to three amino acids have been considered which are shown to be interacting with the ligands observed in the docking studies to determine the hydrogen bonding strengths. The calculated binding energies for the ligands, Ligand**19**, Ligand**8**, Ligand**7**, and Ligand**20**, are −69.39, −49.09, −25.11, and −8.63 kcal/mol, respectively. The N-H⋯O, O-H⋯N, and O-H⋯O type of hydrogen bonds have been observed in the ligand(s)-amino acids complexes. All the hydrogen bond lengths are observed to be around 2.0 Å which shows the stability of the complexes (Table 6). The optimized structure of Ligand**19** has been shown in Figure 8. From Figure 9, it has been observed that the 2-oxobutanoyl group in Ligand**19** has not participated in the amino acid interactions. In Figure 10, the frontier molecular orbital diagrams, the HOMO structure of Ligand**19** shows that the electrons are localized on the central part of the ligand and are not localized on the 2-oxobutanoyl group. The LUMO structure shows that the electrons are localized at phenol and 2-oxobutanoyl groups only. From the HOMO and LUMO structures it can be concluded that the indole, phenol, and benzimidazole units in Ligand**19** are readily available to donate the electrons to the interacting groups of amino acids in the binding site of an enzyme. The HOMO results show that Ligand**19** has the highest HOMO level energy among the ligands. The results of DFT studies are in good agreement with the docking results and show that Ligand**19** is the best inhibitor in the study. The HOMO-LUMO gap, in Ligand**19**, has been observed to be 2.86 kcal/mol. The lowest HOMO-LUMO gap in Ligand**19** among the considered ligands is showing that HOMO of the inhibitor (Ligand**19**) may transfer its electrons to less energy, LUMO, of amino acids residues in the active site of an enzyme. The HOMO-LUMO gaps for the other ligands like Ligand**20**, Ligand**8,** and Ligand**7** have been observed to be 3.42, 3.75, and 4.69 kcal/mol, respectively, given in Table 7.

The large hetero atom containing compounds have shown higher IC_50_ value experimentally in the literature [46, 47]. Electron donor groups can be identified as having more electron density observed from the HOMO picture of a ligand. In Ligand**19** the HOMO is scattered over from phenol to indole group and LUMO is scattered over from phenol to 2-oxo-butanoyl group (Figure 10). The docking results also show that Ligand**19** is involved in the important interactions with the key residues of protein. In Figure S7 (at Supplementary Material available online at http://dx.doi.org/10.1155/2016/7264080), for Ligand**8**, the HOMO is scattered over from the furan to the benzimidazole group and LUMO is scattered only on the 1-chloro-2-nitro-imidazole group. In Figure S3, for Ligand**7**, the HOMO and LUMO are scattered over on the same place of the functional groups. As shown in Figure S11, for Ligand**20,** the HOMO is scattered from the quinoline group to bromobenzene, but the LUMO is scattered from the azo group to bromobenzene. Qualitatively, in Ligand**19** the electron localization between HOMO and LUMO has varied between the functional groups quit higher than the rest of the compounds considered for the DFT study. But, the HOMO-LUMO gap is observed to be smaller in Ligand**19** compared to the other ligands. The results of orbital energies are possibly associated with the charge transfer, *π*⋯*π*, or *π*⋯*σ* stacking between inhibitors and amino acid residues in the binding site of an enzyme.

**Figure 11:**
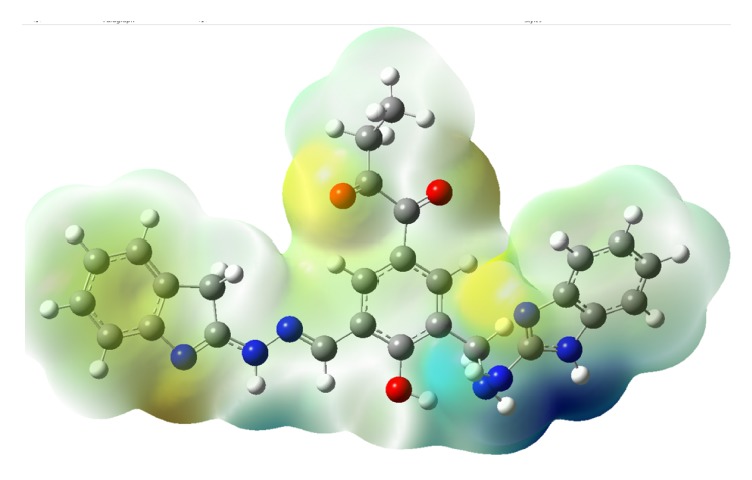
Electrostatic potential maps (ESP) of Ligand**19** at M06/6-31g(d) level in gas phase (scale: −0.867*∗*10^−2^ to 8.67*∗*10^−2^).

The electrostatic potential (ESP) structure for Ligand**19** is shown in Figure 11. The ESP maps are scaled from −0.867*∗*10^−2^ to 0.867*∗*10^−2^. The blue region is observed to be donor and yellow region is observed to be an acceptor. Ligand**19** has more electropositive (blue color) and more electronegative (yellow color) units. In Ligand**19** the benzimidazole and azo groups are revealed to be a hydrogen bond donor from ESP structure which is consistent with the docking results. The benzimidazole group acts as a donor while interacting with the phenylalanine. Similarly, the nitrogen in indole group acts as a hydrogen bond acceptor which is also consistent with the docking results. The indole group is accepting a proton from the aspartic acid as a hydrogen bond acceptor. The molecular electrostatic potential and orbital energies have the feature of being successfully employed as a 3D structural query for virtual screening of databases to identify the potential inhibitors.

## 11. Lipinski Rule of 5

The screening of compounds has been performed through molecular docking approach. The properties of the ligands are examined using using Qikprop, Schrodinger LLC [48]. The property influences the pharmacological activity derived from the Absorption, Distribution, Metabolism, and Excretion to estimate their drug likeliness of the compound. The binding between the ligand and protein is considered with the number of rotatable bonds; the value was determined as <10 which influences the conformational changes [16]. The lowest degree of Log *P* value indicates the good water solubility according to Lipinski's rule. The property seems to be more significant and forecasts the physicochemical property of the drugs carried out in the part of drug designing [49]. Lipinski rule of 5 (RO5) parameters is satisfied by Ligand**8** and Ligand**19**. In order to consider the obtained ligands with minimal acceptors Ligand**19** seems to be chosen as a drug target and is reported in Table 8.

According to Nguyen et al., compound** 2** (here, Ligand**8**) was the better ligand from biological significance and was assessed using IC_50_ values through the* in vitro* and screening studies. Furthermore, the efficiency of structure-based drug designing approach has great impact on investigating the series of compounds. The scoring of molecular mechanics (MMGBSA) based approach strengthens the scoring functions when comparing to the docking analysis. The scoring value has shown the correlation between the computed score and experimental data. It can be considered as reliable approach to handle more structurally dissimilar ligands [9]. In the present study, remarkable results are obtained with this methodology when compared to docking scoring function; the MMGBSA procedure provided more better association between calculated binding free energies and biological activity (Tables 3 and 4), extended to MD simulation and DFT approaches. The study exhibits the significance of the set of inhibitors identified in the order of binding energy, hydrogen bond interactions, gas phase energy values, and physicochemical properties. Ligand**19** with the experimental activity of 1.3 *μ*M (Table 1) has shown the significant activity in terms of hydrogen bond interactions with His51, Asp75, Gly133, Phe130, Gly151, and Gly153 and determines the high affinity suggesting a possible mechanism of the action compared to Ligand**07** and Ligand**08**. The current investigation has a good agreement with the earlier reports. Moreover, the molecular modelling approaches enhanced to swift identification and initial optimization of novel series of inhibitors for the NS2B/NS3 proteins.

## 12. Conclusions

In this study, the novel Ligand**19** termed as an inhibitor for the dengue virus NS2B/NS3 protease enzyme was interpreted from the series of docking and molecular dynamics simulation protocols. Ligand**19** seems to be more potent than Ligand**8** and Ligand**7** from the outcome of docking, dynamics, DFT, and physicochemical properties analysis. The six residues of the Wild Type (WT) protein were mutated with alanine; the simulation analysis reveals that it may disrupt their stability and leads to changing their conformation. Moreover, the FMO of DFT studies revealed the lowest HOMO-LUMO for Ligand**19** reported as a best inhibitor in the study. Our DFT results of binding energy, FMO and ESP, are in good agreement with the docking studies qualitatively and quantitatively. The biological activity of the identified ligand seems to be more significant according to Lipinski's rule of 5 (RO5). The results obtained from this analysis will be sufficient for the further investigations.

## Supplementary Material

Figure S1: Optimized structure of Ligand 7 at M06/6-31g(d) level in gas phase.
Figure S2: Optimized structure of Ligand 7 with Isoleucine (hydrogen bond length: 1.997 Å) and Glycine (hydrogen bond length: 1.805 Å) at M06/6-31g(d) level in gas phase.
Figure S3: Molecular orbital distribution plots of HOMO and LUMO states in the ground state of the Ligand 7 in gas phase.
Figure S4: Electrostatic potential maps (ESP) of Ligand 7 at M06/6-31g(d) level in gas phase. (Scale: −0.867 *∗* 10^−2^ to 8.67 *∗* 10^−2^)
Figure S5: Optimized structure of Ligand 8 at M06/6-31g(d) level in gas phase.
Figure S6: Optimized structure of Ligand 8 with Phenyl alanine (hydrogen bond length: 2.392 and 1.874 Å) and Tyrosine (hydrogen bond length: 1.746 Å) at M06/6-31g(d) level in gas phase.
Figure S7: Molecular orbital distribution plots of (a) HOMO and (b) LUMO states in the ground state of the Ligand 8 in gas phase.
Figure S8: Electrostatic potential maps (ESP) of Ligand 8 at M06/6-31g(d) level in gas phase. (Scale: −0.867 *∗* 10^−2^ to 8.67 *∗* 10^−2^).
Figure S9: Optimized structure of Ligand 20 at M06/6-31g(d) level in gas phase.
Figure S10: Optimized structure of Ligand 20 with Glycine (hydrogen bond length: 1.968 and 2.417 Å) at M06/6-31g(d) level in gas phase.
Figure S11: Molecular orbital distribution plots of (a) HOMO and (b) LUMO states in the ground state of the Ligand 20 in gas phase.
Figure S12: Electrostatic potential maps (ESP) of Ligand 20 at M06/6-31g(d) level in gas phase. (Scale: −0.867 *∗* 10^−2^ to 8.67 *∗* 10^−2^).

## Figures and Tables

**Table 1:** GLIDE-XP energies (kcal/mol) of selected 20 ligands with IC_50_ values.

Sl. number	Ligand	Source	Energy	IC_50_ (*µ*M)
1	Ligand**1**	PubChem	−34.18	3.0
2	Ligand**2**	PubChem	−36.51	1.29
3	Ligand**3**	PubChem	−43.10	1.29
4	Ligand**4**	PubChem	−46.75	1.29
5	Ligand**5**	PubChem	−38.16	1.29
6	Ligand**6**	PubChem	−44.67	33.89
7	Ligand**7**	PubChem	−45.83	0.1
8	Ligand**8**	ChemDiv	−48.40	15.6
9	Ligand**9**	ChemDiv	−44.21	86.7
10	Ligand**10**	ChemDiv	−31.34	32.2
11	Ligand**11**	ChemDiv	−41.67	12.5
12	Ligand**12**	ChemDiv	−39.14	3.9
13	Ligand**13**	ChemDiv	−39.09	26.4
14	Ligand**14**	ChemDiv	−51.31	75.3
15	Ligand**15**	PubChem	−42.52	0.12
16	Ligand**16**	PubChem	−31.57	4.7
17	Ligand**17**	PubChem	−35.18	1.08
18	Ligand**18**	PubChem	−36.41	0.009
19	Ligand**19**	PubChem/DrugBank	−41.00	1.37
20	Ligand**20**	PubChem/DrugBank	−41.16	1.37

**Table 2:** Induced Fit Docking results.

Sl. number	Ligand	Energy
1	Ligand**7**	−49.17
2	Ligand**8**	−62.71
3	Ligand**19** (pose 1)	−47.00
4	Ligand**19** (pose 2)	−64.16

**Table 3:** The best poses and their hydrogen bond distance in Å.

Ligands	Hydrogen bond interactions	Distance (Å)
Ligand**7**	Gly151 O⋯H-O (Lig)	2.29
	Ile36 O⋯H-O (Lig)	2.00

Ligand**8**	Lig N-H⋯O Phe130	1.72
	Tyr150 O⋯H-O Lig	2.16
	Gly153 N-H⋯N Lig	2.41

Ligand**19**—pose 1	Lig N-H⋯O Phe130	2.09
	Gly151 O⋯H-N (Lig)	2.03
	Lig N-H⋯O Asp75	1.81

Ligand**19**—pose 2	Gly151 O⋯H-O Lig	2.03
	Lig N-H⋯O Gly153	2.48
	Lig N-H⋯O Asp75	2.43
	Lig N-H⋯H Asp75	2.44
	Lig N-H⋯O Phe130	2.06

**Table 4:** Binding free energies derived from MM-GBSA calculations using single protein-ligand complexes (unit: kcal/mol).

Sl. number	Ligand	Δ*G*			Δ*G*_sol_	Δ*G*_lipo_	Δ*G*_bind_
		Δ*G*_coul_	Δ*G*_vdw_	Δ*G*_cov⁡_	Δ*G*_solGA_		
1	Ligand**7**	−20.706	−42.822	6.453	24.162	−20.698	−53.611
2	Ligand**8**	−24.938	−37.107	2.206	16.482	−28.798	−72.155
3	Ligand**19**	−26.933	−42.448	1.552	27.567	−34.550	−74.812

**Table 5:** Comparison with existing ligands.

Residues	4-Hydroxypanduratin A	Panduratin A	Ligand **19**	Ligand **8**	Ligand **7**
Ile36					√
His51			√		
Asp75	√		√		
Gly133			√		
Phe130			√	√	
Gly151	√	√	√		√
Gly153			√	√	

**Table 6:** The binding energies are calculated at M06/6-31g(d) level of theory in gas phase for the ligand-amino acid complexes.

System	Binding energy (kcal/mol)
Ligand19-Gly-Asp-Phe	−69.39
7-Ile-Gly	−25.11
8-Phe-Tyr	−49.09
20-Gly	−8.63

**Table 7:** Frontier Molecular Orbital Energies of optimized structures of Ligands**19**, **7**, **8**, and **20** at M06/6-31g(d) level in gas phase. Highest Occupies Molecular Orbital (HOMO)^a^, Lowest Unoccupied Molecular Orbital (LUMO)^b^, and HOMO-LUMO gap (HLG)^c^.

System	HOMO^a^ (eV)	LUMO^b^ (eV)	HLG^c^ (kcal/mol)
Ligand**19**	−0.199	−0.0751	2.86
Ligand**7**	−0.2334	−0.0299	4.69
Ligand**8**	−0.2287	−0.0656	3.76
Ligand**20**	−0.2192	−0.0707	3.42

**Table 8:** Properties of ligand molecules.

Sl. number	Ligands	Mol.Wt	log⁡*P*	Hydrogen bond acceptor	Hydrogen bond donor	Free rotatable bonds
1	Ligand**7**	821.00	4.21	15	6	12
2	Ligand**8**	499.95	3.12	11	1	9
3	Ligand**19**	495.53	3.83	10	5	9
