# Supplementary material for: In Silico Screening, Alanine Mutation, and DFT Approaches for Identification of NS2B/NS3 Protease Inhibitors
Source: Biochem Res Int. 2016 Feb 25;2016:7264080. doi: 10.1155/2016/7264080 (PMC4785246; doi:10.1155/2016/7264080)
Supplement: Supplementary file 1 — Figure S1: Optimized structure of Ligand 7 at M06/6-31g(d) level in gas phase. Figure S2: Optimized structure of Ligand 7 with Isoleucine (hydrogen bond length: 1.997 Å) and Glycine (hydrogen bond length: 1.805 Å) at M06/6-31g(d) level in gas phase. Figure S3: Molecular orbital distribution plots of HOMO and LUMO states in the ground state of the Ligand 7 in gas phase. Figure S4: Electrostatic potential maps (ESP) of Ligand 7 at M06/6-31g(d) level in gas phase. (Scale: −0.867 ∗ 10−2 to 8.67 ∗ 10−2) Figure S5: Optimized structure of Ligand 8 at M06/6-31g(d) level in gas phase. Figure S6: Optimized structure of Ligand 8 with Phenyl alanine (hydrogen bond length: 2.392 and 1.874 Å) and Tyrosine (hydrogen bond length: 1.746 Å) at M06/6-31g(d) level in gas phase. Figure S7: Molecular orbital distribution plots of (a) HOMO and (b) LUMO states in the ground state of the Ligand 8 in gas phase. Figure S8: Electrostatic potential maps (ESP) of Ligand 8 at M06/6-31g(d) level in gas phase. (Scale: −0.867 ∗ 10−2 to 8.67 ∗ 10−2). Figure S9: Optimized structure of Ligand 20 at M06/6-31g(d) level in gas phase. Figure S10: Optimized structure of Ligand 20 with Glycine (hydrogen bond length: 1.968 and 2.417 Å) at M06/6-31g(d) level in gas phase. Figure S11: Molecular orbital distribution plots of (a) HOMO and (b) LUMO states in the ground state of the Ligand 20 in gas phase. Figure S12: Electrostatic potential maps (ESP) of Ligand 20 at M06/6-31g(d) level in gas phase. (Scale: −0.867 ∗ 10−2 to 8.67 ∗ 10−2). [file 7264080.f1.docx]

***in silico* analysis, Alanine and DFT approaches for identification of NS2B/NS3 Protease inhibitors.**

Balajee R*^1^, V. Srinivasadesikan^2,^ Sakthivadivel M^3^,Gunasekaran P^3^

^1^ Medicinal Chemistry Group, Institute of Chemistry of Sao Carlos, University of Sao Paulo, Sao Carlos, Brazil

^2^ Department of Applied Chemistry, National Chiao Tung University, Hsinchu, Taiwan

^3^ King Institute of Preventive Medicine, Guindy, Chennai, India

***Corresponding Author:** rbalajeebio@gmail.com


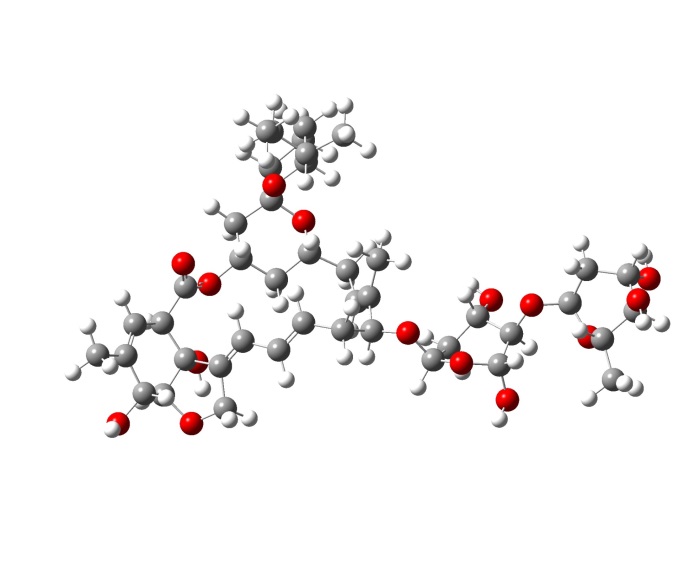


Figure S1: Optimized structure of Ligand 7 at M06/6-31g(d) level in gas phase.


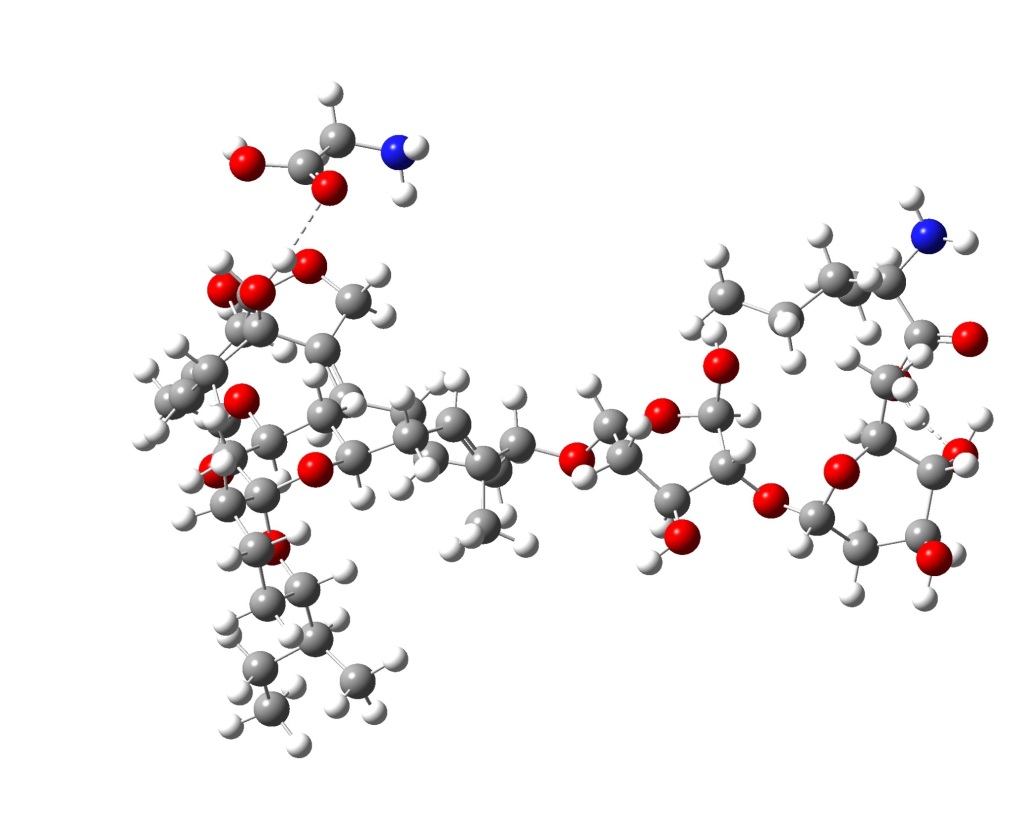


**1.997**

**1.805**

Figure S2: Optimized structure of Ligand 7 with Isoleucine (hydrogen bond length: 1.997 Å) and Glycine (hydrogen bond length: 1.805 Å) at M06/6-31g(d) level in gas phase.


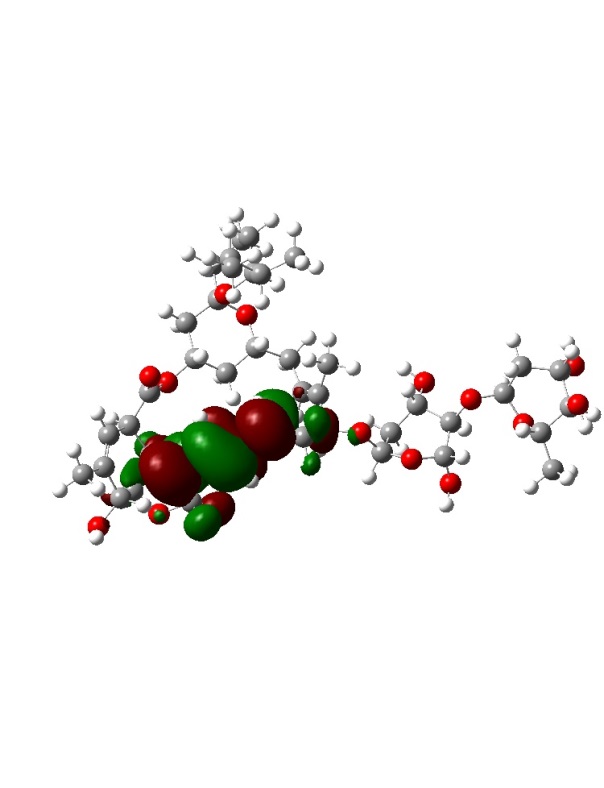


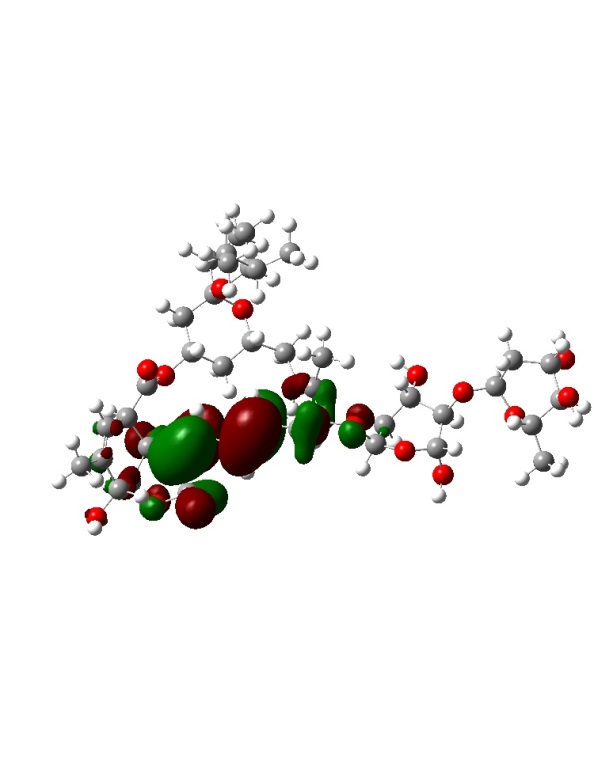


Figure S3: Molecular orbital distribution plots of HOMO and LUMO states in the ground state of the Ligand 7 in gas phase.


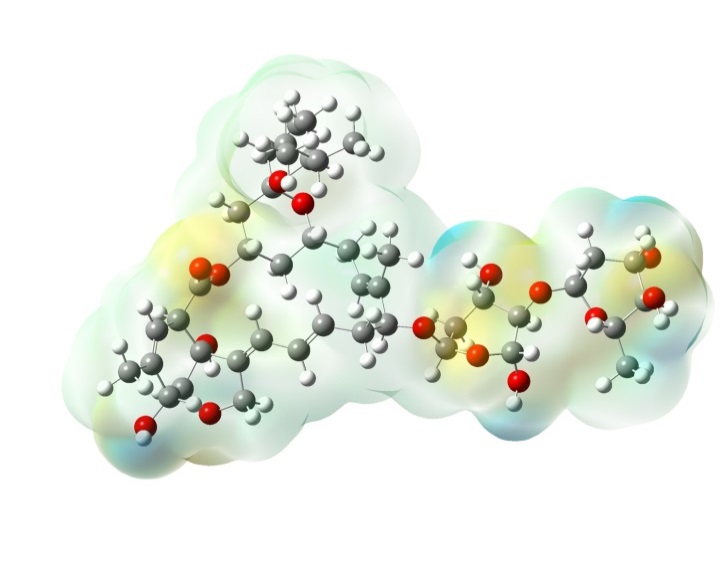


Figure S4: Electrostatic potential maps (ESP) of Ligand 7 at M06/6-31g(d) level in gas phase. (Scale: -0.867*10^-2^ to 8.67*10^-2^)


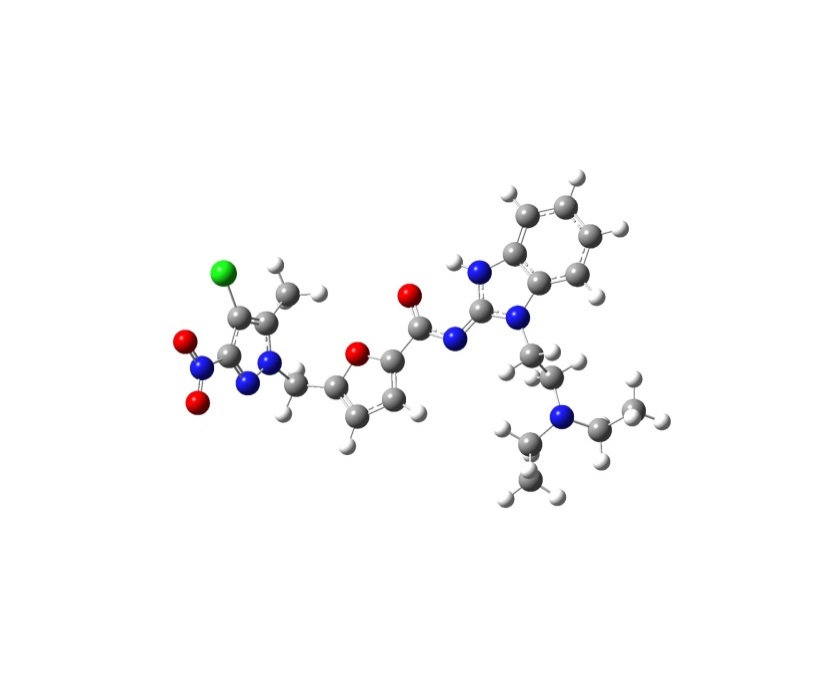


Figure S5: Optimized structure of Ligand 8 at M06/6-31g(d) level in gas phase.


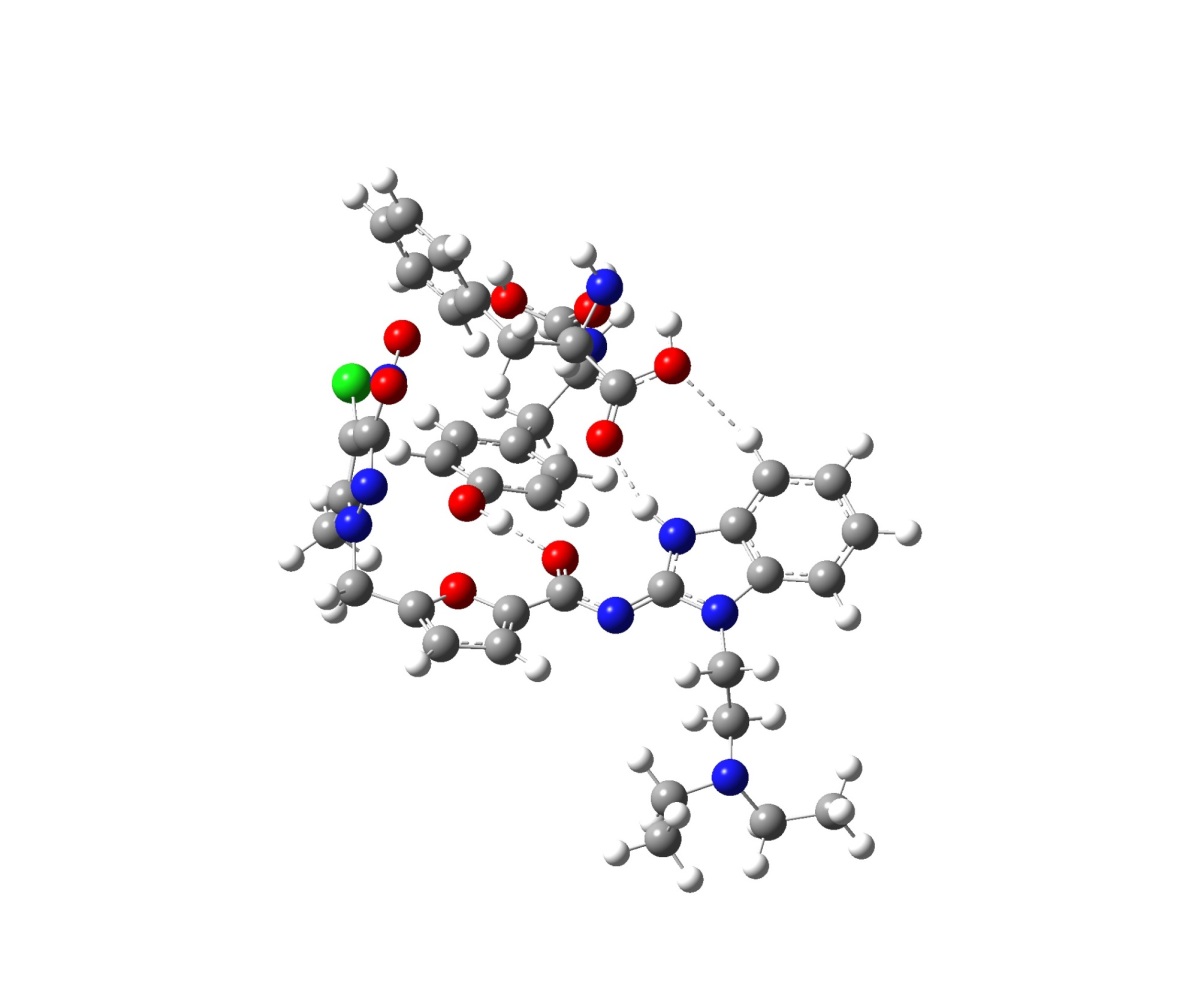


**2.392**

**1.874**

**1.746**

Figure S6: Optimized structure of Ligand 8 with Phenyl alanine (hydrogen bond length: 2.392 and 1.874 Å) and Tyrosine (hydrogen bond length: 1.746 Å) at M06/6-31g(d) level in gas phase.

(a)


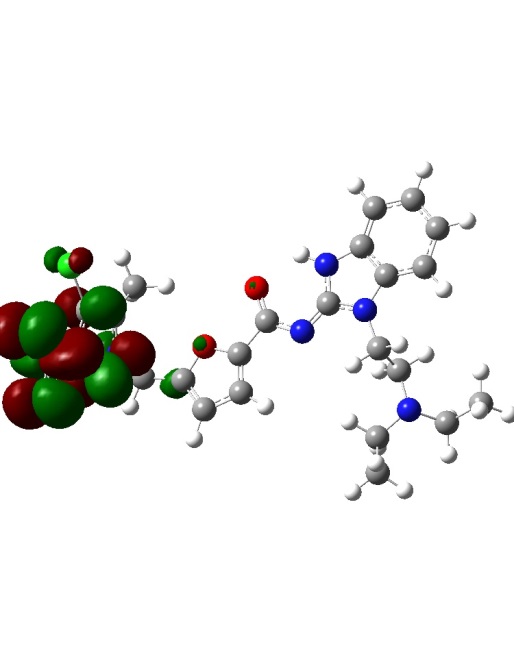

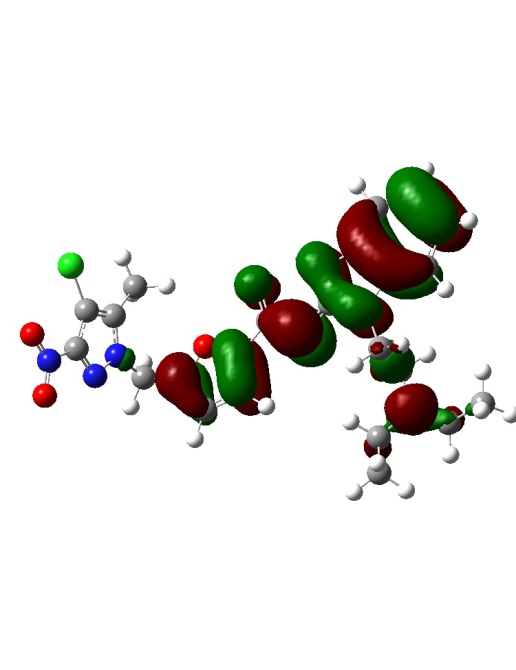
(b)

Figure S7: Molecular orbital distribution plots of (a) HOMO and (b) LUMO states in the ground state of the Ligand 8 in gas phase.


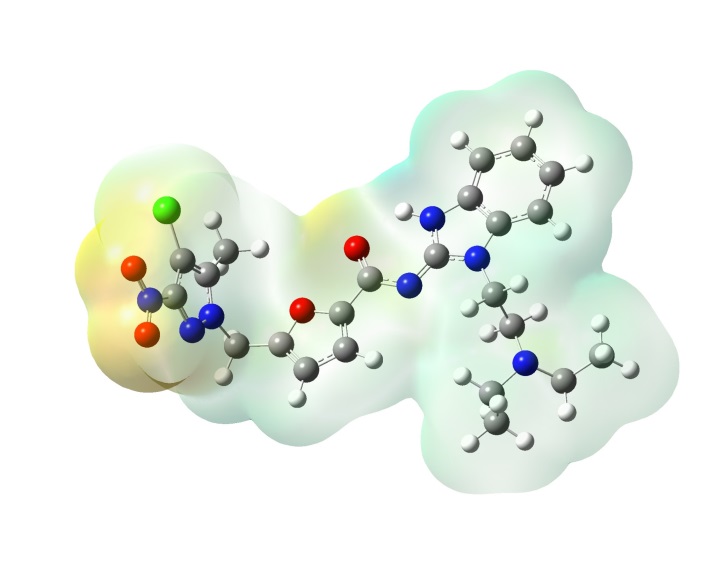


Figure S8: Electrostatic potential maps (ESP) of Ligand 8 at M06/6-31g(d) level in gas phase. (Scale: -0.867*10^-2^ to 8.67*10^-2^).


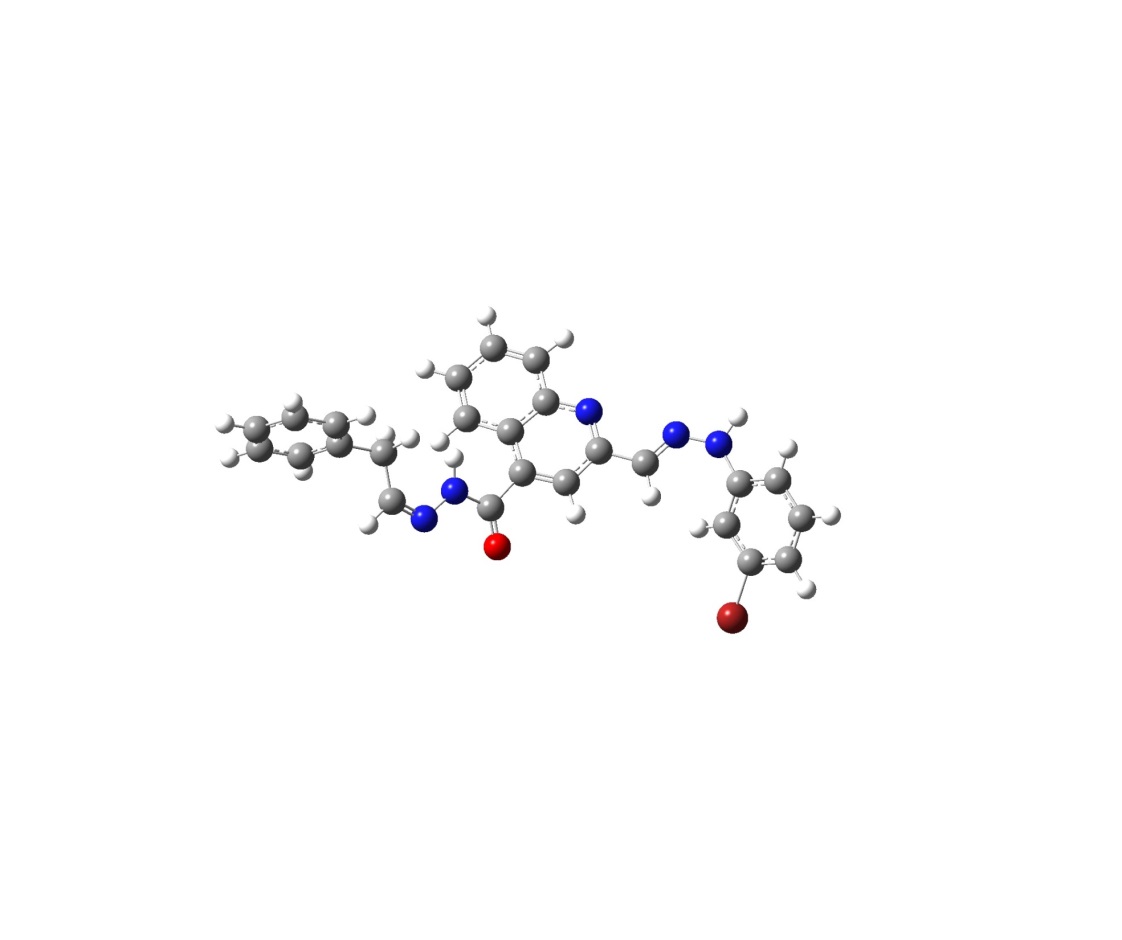


Figure S9: Optimized structure of Ligand 20 at M06/6-31g(d) level in gas phase.


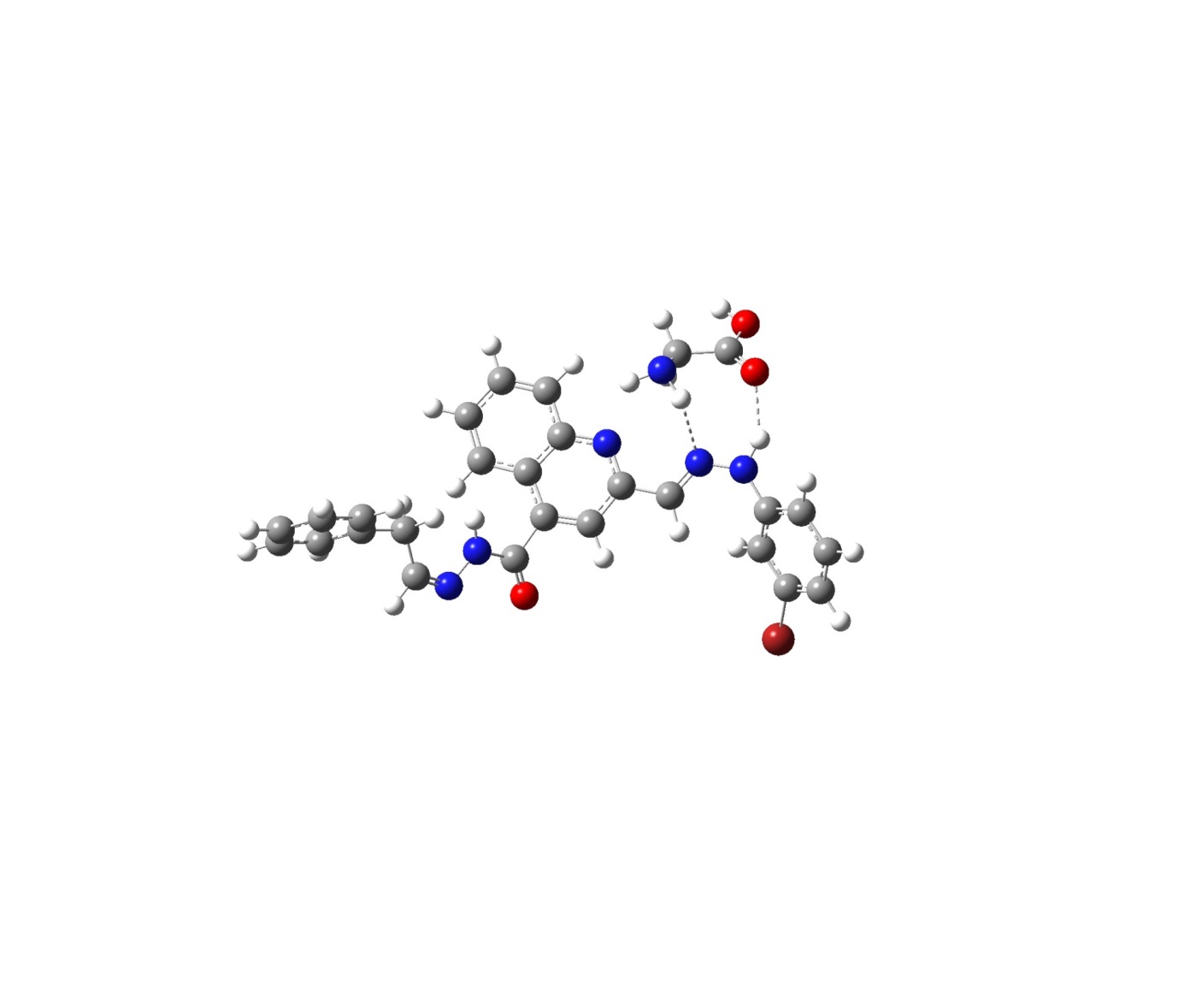


**1.968**

**2.417**

Figure S10: Optimized structure of Ligand 20 with Glycine (hydrogen bond length: 1.968 and 2.417 Å) at M06/6-31g(d) level in gas phase.


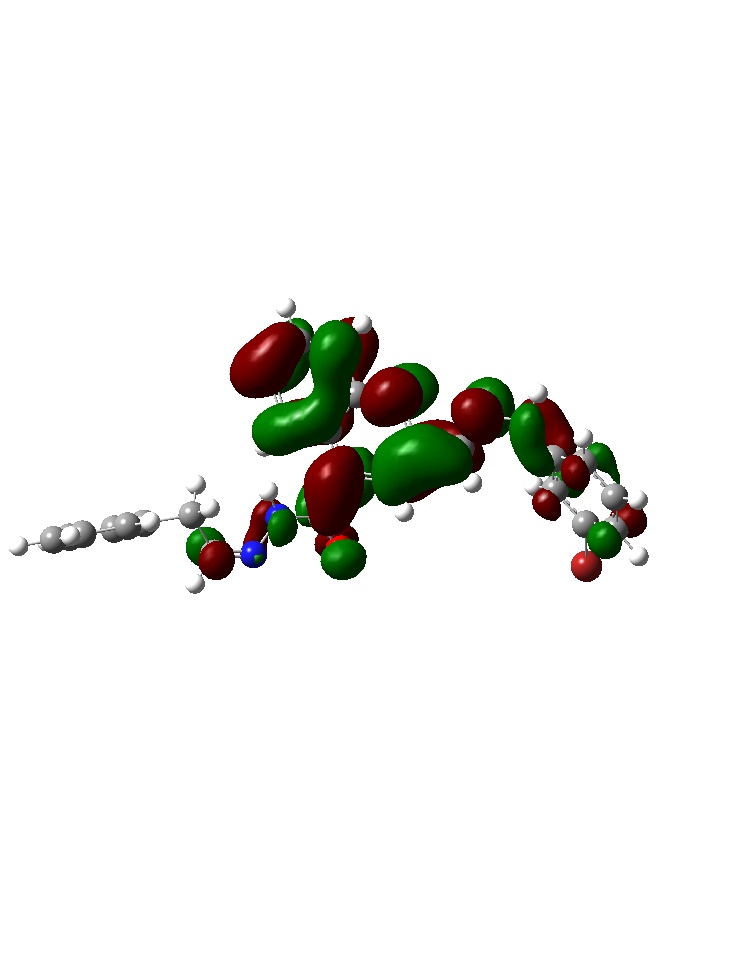

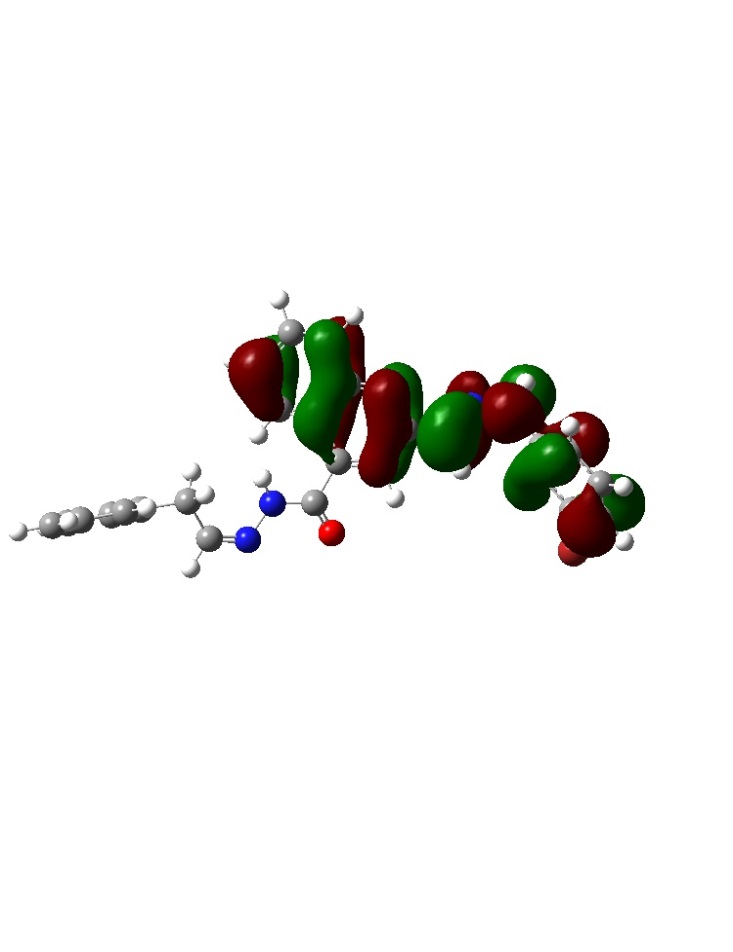


Figure S11: Molecular orbital distribution plots of (a) HOMO and (b) LUMO states in the ground state of the Ligand 20 in gas phase.


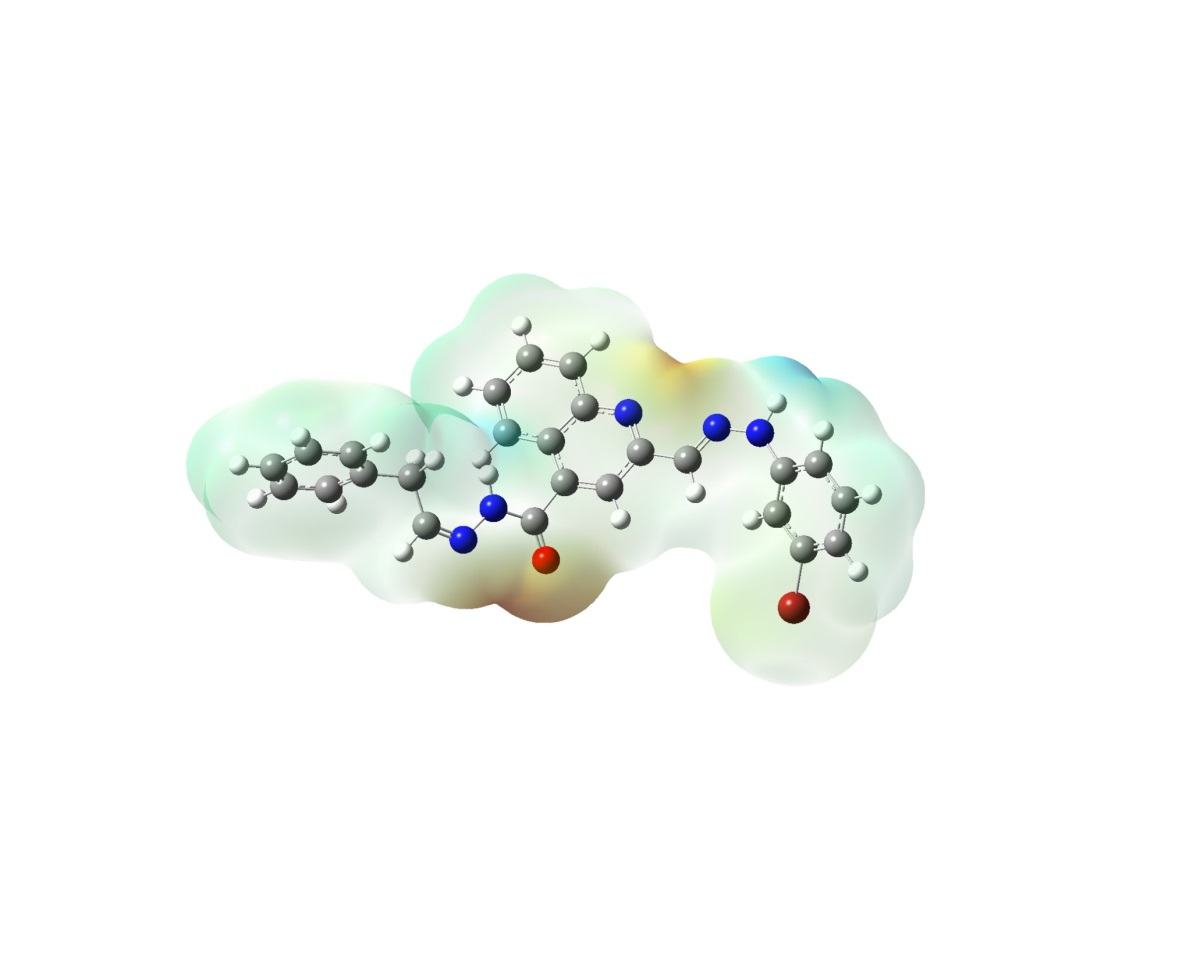


Figure S12: Electrostatic potential maps (ESP) of Ligand 20 at M06/6-31g(d) level in gas phase. (Scale: -0.867*10^-2^ to 8.67*10^-2^).
